# Developing an Inclusive Dance Guide for Children With Cerebral Palsy: A Co‐Design Process and Initial Feasibility Study

**DOI:** 10.1111/hex.70304

**Published:** 2025-05-27

**Authors:** Eduardo Duarte Machado, Laura Miller, Joanna Nicholas, Joanne Cross, Rhyannon Orr, Michael H. Cole

**Affiliations:** ^1^ Development and Disability Over the Lifespan, Healthy Brain and Mind Research Centre Australian Catholic University Brisbane Australia; ^2^ Faculty of Health Science, School of Allied Health Australian Catholic University Brisbane Australia; ^3^ Western Australian Academy of Performing Arts Edith Cowan University Mount Lawley Australia; ^4^ Family Advocacy Hornsby New South Wales Australia

**Keywords:** cerebral palsy, co‐design, inclusive dance

## Abstract

**Background:**

Participation in community activities has shown positive outcomes for all children, yet those with cerebral palsy (CP) still experience varying levels of inclusion. This study aimed to address the challenges faced by families of children with CP in accessing community‐based dance classes by co‐designing a practical guide to support their inclusion.

**Design:**

The guide was developed through a collaborative process involving academic researchers, dance professionals, therapists and families of children with CP. The co‐design phase was guided by the International Association for Public Participation (IAP2), workshops were conducted with 11 research partners, including two parents of children with CP, two community dance teachers, two therapists with experience in dance interventions and five academic researchers. Feedback from ‘Dance Teacher Reviewers’ who were not involved in the co‐design was also incorporated to support the guide's initial feasibility.

**Results:**

The collaborative efforts resulted in an evidence‐based guide shaped by lived experiences. It provides actionable strategies to implement inclusive dance approaches effectively, supporting the inclusion of children with CP in community dance settings.

**Conclusion:**

The co‐designed guide represents a significant step toward facilitating inclusive dance classes for children with CP. Future research should explore the guide's effectiveness and feasibility of implementation across various community settings to ensure its broader applicability and impact.

**Patient or Public Contribution:**

Parents of children with CP, dance teachers and therapists actively contributed to all stages of this study, from public consultation to collaborative co‐design workshops, decision‐making, review and refinement of the guide.

## 1 Background

Regular participation in physical activity has consistently been associated with positive physical, social and emotional outcomes [1, 2]. However, for children with cerebral palsy (CP), achieving these outcomes often requires careful consideration of their unique needs and individualised adaptations [3, 4]. Dance has emerged as a promising activity to promote physical activity, social inclusion and community connection [5, 6]. For children with CP, inclusive dance provides opportunities to challenge societal perceptions of disability and fosters an environment where children with and without disabilities can learn together [7, 8]. Furthermore, families are increasingly seeking community‐based activities such as dance that prioritise meaningful participation with peers over segregation or therapy‐focused integration [9, 10].

Despite growing evidence supporting the benefits of dance for children with CP, there remain significant barriers to full participation and inclusion [11, 12]. These include, for example, ableist attitudes, inaccessible facilities and limited training for dance teachers [11, 13]. A recent systematic review mapped participation outcomes of dance to the Family of Participation‐Related Constructs (fPRC), highlighting that dance can positively influence both intrinsic factors (activity competence, sense of self and preferences) and extrinsic factors (person‐environment interactions) for people with CP [6, 14]. However, to achieve meaningful, participation‐focused outcomes, it was recommended that rehabilitation professionals should collaborate and partner with knowledge users to understand and address their goals and challenges when participating in dance [6]. This effort includes developing training programs for dance teachers that emphasise strategies to promote a deeper understanding of the unique needs of children with CP [12, 15].

To date, there has been limited involvement of knowledge users, including children with CP, their parents and caregivers, in the development of inclusive dance classes that focus on participation, particularly in community settings [6, 16], despite evidence demonstrating its importance for improved and impactful outcomes [17, 18]. Authentic knowledge user engagement in research that empowers knowledge users as research partners has become increasingly recognised as essential for ensuring that research outcomes are relevant, effective and reflective of their needs [19, 20]. Such collaboration helps ensure strategies for inclusion are evidence‐based, sustainable and adaptable to real‐world contexts [20, 21]. This participatory approach represents a shift from traditional models, where children and their families assumed passive roles in research, to active roles as partners in the study design, implementation and dissemination, thereby enhancing the relevance and impact of the research [17, 18, 22].

This article provides a detailed description of the co‐design process undertaken to develop an inclusive dance approach guide. The guide aims to support the inclusion of children with CP in community‐based dance classes so they can participate with their peers. The co‐design process was structured in three key stages: sequential focus groups, iterative co‐design workshops and initial feasibility appraisal of the guide's content. This process emphasised the importance of creating a genuine partnership, enabling participants to contribute at different levels of the International Association for Public Participation (IAP2), as decided by them, with the team committed to equally hearing, respecting, and incorporating everyone's contributions throughout the design of the guide [23, 24]. This approach aligns with co‐design principles, which aim to democratise research and ensure outputs reflect participants' needs and experiences [23, 25]. The aims of this study were to (i) co‐design a practical guide to support the inclusion of children with CP in community‐based dance classes; and (ii) explore the initial feasibility of the guide's content, focusing on its acceptability, demand and sensibility, through independent external user consultation.

## 2 Methodology

### 2.1 Definitions

In this study, the term ‘knowledge user’ (also referred to as end‐user, consumers, stakeholder or PPI in other frameworks) refers to children with CP, their parents/caregivers, service providers and dance/physical education teachers involved in community or school dance classes. This term broadens the scope and role of participants, to encompass a diverse range of stakeholders [26]. Moreover, in line with the social model of disability, children with CP are referred to as dancers throughout the guide, emphasising their active participation and identity as a dancer [27].

The authors also acknowledge the inconsistency across academia, nongovernment organisations and civil society regarding the definition of inclusion [10, 28]. Inclusion is frequently confused with integration and community presence, which primarily focus on physical location rather than meaningful engagement [28]. Many studies use these terms interchangeably without providing clear definitions, which contributes to ongoing confusion about what constitutes true inclusion [10]. For the purposes of this study, inclusion is defined as creating an environment where children with disabilities can fully and actively participate alongside their peers, with the necessary modifications and supports that ensure meaningful engagement, fostering a sense of belonging and equitable access to opportunities [10, 29].

### 2.2 Overview

Participatory action research (PAR) was the overarching methodology used to facilitate evidence‐based co‐development of the ‘Co‐Designed Inclusive Dance Approach Guide – Inclusion of Children with Cerebral Palsy’ [30, 31]. PAR focuses on authentic partnership and collaboration, celebrating the experiences and efforts of democratic peer relationships where participants affirm their right and ability to have a say in decision‐making processes [30]. Specifically, we employed *Integrated Knowledge Translation* (iKT) [32] and *Partnership‐focused Principles‐driven Online Co‐design* (P‐POD) [23], two collaborative approaches that integrate both researchers and knowledge users as integral members of the research team to co‐create, implement and evaluate projects [23, 32]. This study was also guided by the International Association for Public Participation (IAP2) *Spectrum of Public Participation* model, which aims to improve public participation in research and presents a tangible and practical scale with the pillar of public engagement [21, 33].

**Figure 1:**
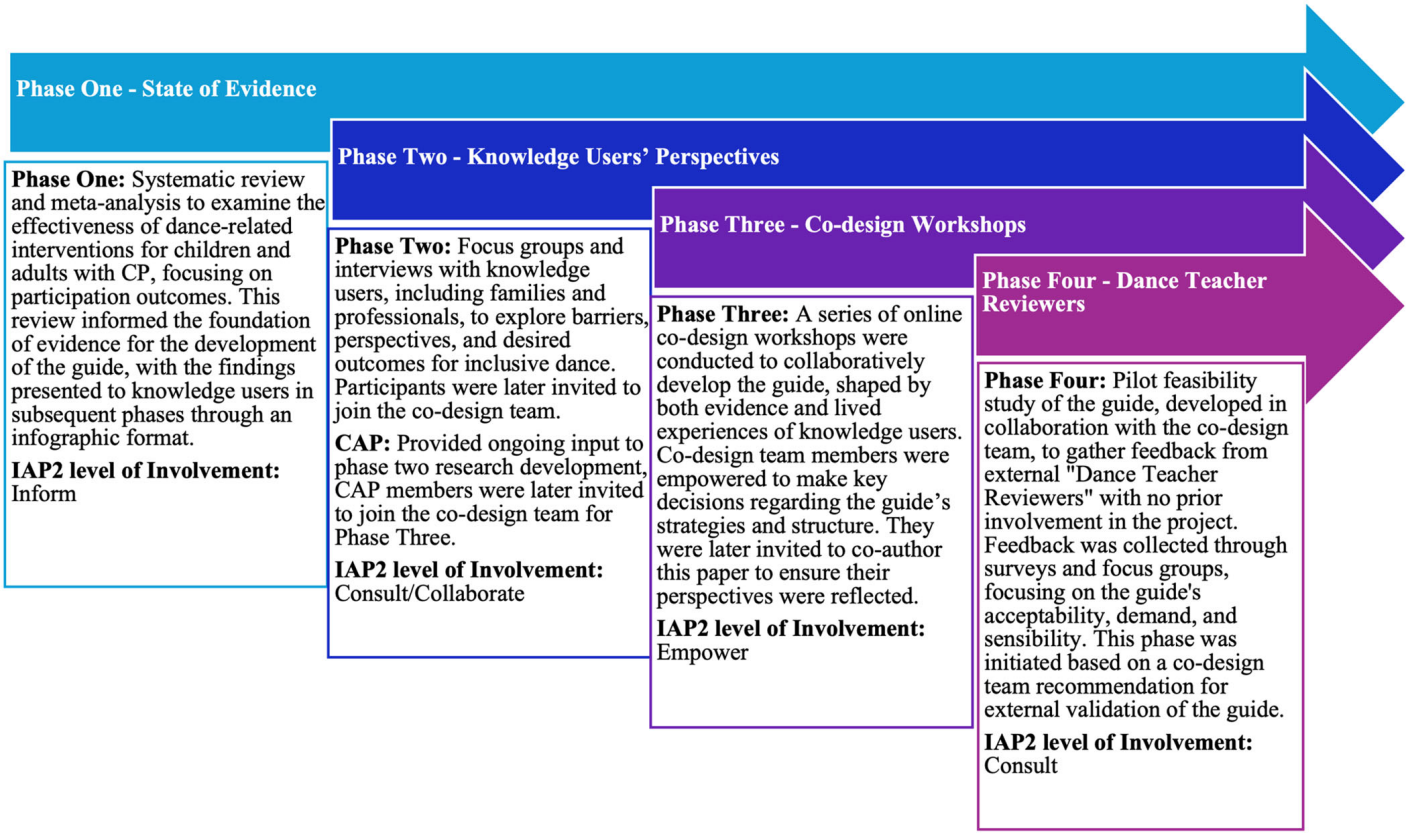
Multiphase and iterative co‐design process: knowledge user involvement in each phase and its level of engagement on the IAP2 Spectrum of Public Participation.

The guide was developed through a multiphase, iterative co‐design process (Figure 1), initially informed by a recently published systematic review (Phase 1) [6]. Phase 2 involved a Consumer Advisory Panel (CAP) and public consultations. The CAP provided ongoing input to research development, including recruitment strategies, data collection questions, data analysis and outputs for publication (currently under review). Focus groups and interview participants included (i) children with CP aged 7 and older, (ii) their families, (iii) parents of children under 7, (iv) dance teachers and (v) health professionals. The perspectives and lived experiences of this diverse group of knowledge users, together with peer reviewed evidence, informed the strategies and resources developed in the co‐design workshops in Phase 3. Details of the CAP's structure and role are outlined in the GRIPP‐2 form (Appendix S1) [34].

The current paper focuses on Phases 3 and 4 of the co‐design process, which included (i) a series of co‐design workshops to develop the guide, involving five rounds of co‐creation; (ii) internal iterative review by the co‐design team; and (iii) external review of the guide's content by a group of independent ‘Dance Teacher Reviewers’, who had no prior exposure to the guide. This study received ethical approval from the Australian Catholic University's Human Research Ethics Committee (HREC) (Application ID: 2021‐302H).

### 2.3 Establishing a Collaborative Partnership With Knowledge Users

Phase 3: Convenience sampling was used to recruit 10–12 research partners to form the co‐design team. While co‐design and qualitative research have no strict guidelines for sample sizes, the literature suggests that 6–12 participants provide an effective balance [24, 31]. This cohort size supports interactive discussions, allows for subgroup work and provides flexibility to account for potential dropouts, ensuring a balance between depth of collaboration and manageability in co‐design settings [31]. Knowledge users from previous phases (members of the CAP and Phase 2 participants) were invited to collaborate in Phase 3. Although the developed guide primarily targets school‐age children with CP, the involvement of parents of children under 7 and young adults with CP was recommended by the CAP during Phase 2. This broader inclusion aimed to capture lived experiences and diverse perspectives on the barriers and facilitators of raising and supporting a young dancer with CP through childhood into young adulthood.

Phase 4: Five dance teachers from community‐based or school‐based dance classes, who had not participated in any previous phases of the co‐design, including the CAP, were purposively selected to review the guide. Recruitment focused on dance teachers with different levels of experience and from diverse dance genres and teaching areas (e.g., community, private, school‐based), ensuring a broad range of perspectives relevant to the guide's intended implementation [35]. All participants were individually recruited through the networks of the research team and the CAP.

### 2.4 Phase 3 Co‐Design Workshops

Phase 3 consisted of two key parts: orientation and education of the co‐design team, followed by a series of collaborative co‐design workshops. The initial orientation focused on equipping knowledge users and researchers with the necessary tools and frameworks for meaningful engagement, whereas the workshops involved iterative collaboration to co‐design and refine the guide through feedback and discussion.

#### 2.4.1 Orientation and Education

To support research partners in the co‐design process during workshop 1, partners received orientation regarding their involvement in research utilising knowledge translation tools from CanChild's *Family Engagement in Research (FER) program* [36]. The FER program offers tools that use child and family‐friendly language to help participants become familiar with the co‐design process and its objectives [25, 37]. Core tools included the FER Engagement and Self‐Reflection Tool (including goal setting, check‐in and facilitation guide) and the FER: A Conversation Guide. To ensure that these frameworks were implemented effectively, the lead researcher completed the FER Course (https://www.canchild.ca/en/research-in-practice/family-engagement-program/fer-course).

#### 2.4.2 Collaborative Co‐Design Workshops

**Figure 2:**
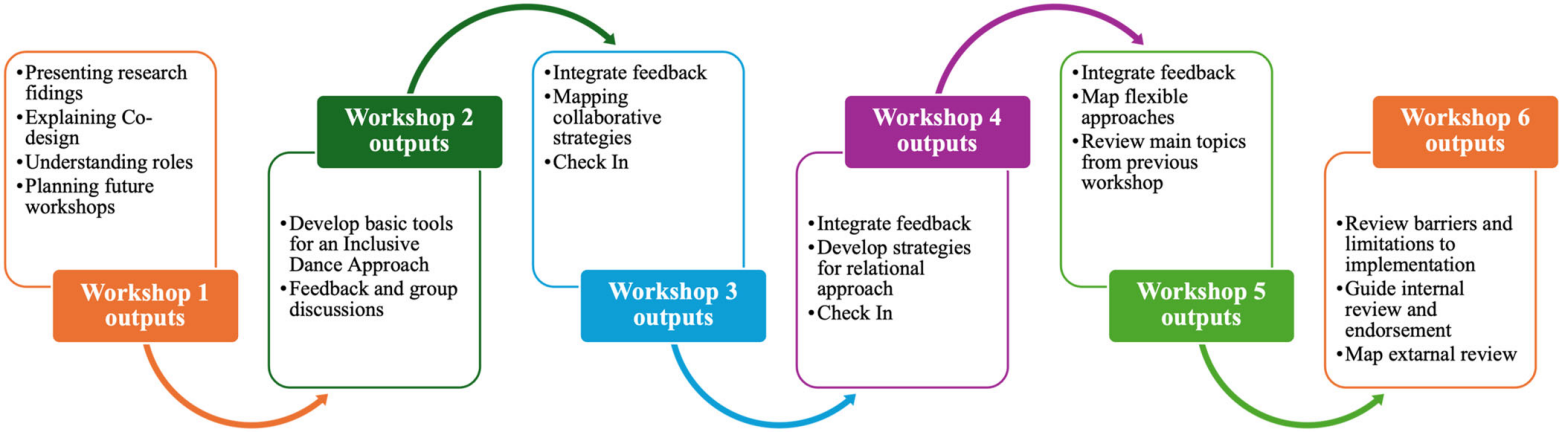
Co‐design workshops process for co‐creation of the guide.

A series of six group‐based co‐design workshops (Figure 2) were conducted online via Zoom to facilitate participation across different schedules and locations. Email exchanges allowed for ongoing collaboration and feedback between sessions, ensuring flexibility. The first workshop discussed the results of the systematic review (Phase 1) and focus groups/interviews (Phase 2). Subsequently, workshops 2–5 mapped the purpose and structure, focusing on identifying and critically reviewing relevant information and resources based on the suggestions, needs and experiences of knowledge users. Strategies were iteratively developed, with discussion and feedback from the co‐design team recorded, considered and integrated into the next workshop.

Following the fifth workshop, the guide underwent an internal review phase where members of the co‐design team were given 1 month to thoroughly review and assess strategies for inclusion, the tools and resources used, and the guide's interface, considering its application in real‐world dance contexts. Feedback was provided via email and during the final workshop to ensure that the perspectives of all team members were accurately reflected.

#### 2.4.3 Co‐Design Engagement Plan

Once the partnership was established, the FER Goal Setting tool was used to discuss project hopes, goals and expectations [38, 39]. A team charter was developed to outline roles and communication channels ensuring clarity and alignment from the outset [18, 40]. The Involvement Matrix provided autonomy to research partners, enabling them to choose diverse roles throughout the co‐design workshops, such as Listener, Co‐thinker, Advisor, Partner and Decision‐maker [41, 42]. Regular check‐ins allowed partners to review their involvement, adjust roles as needed and receive feedback on their contributions [18, 41]. These steps were designed to foster a collaborative environment, balancing power dynamics and ensuring active and meaningful participation from all stakeholders.

Workshop agendas, along with supporting documents and suggested resources, were prepared as facilitation resources for knowledge users to provide sufficient time for reflection, idea generation and planning before each workshop [43]. Questions from the *FER Team Discussion Facilitation Guide* and *FER: A Conversation Guide* were incorporated and adapted throughout the workshops to facilitate group discussion. The success of the working relationship between the researchers and knowledge users was contingent on strong communication and interpersonal skills, as well as facilitation skills [18, 43].

### 2.5 Phase 4 Initial Feasibility of the ‘Co‐Designed Inclusive Dance Approach Guide – Inclusion of Children With Cerebral Palsy’

To explore the guide's initial feasibility, we employed a broader, bottom‐up approach, as recommended by knowledge users, to address practical concerns such as whether the guide would be attractive to community‐based dance teachers or suitable for regular use by people with diverse dance experience and teaching qualifications, questions often overlooked in traditional top‐down approaches [44]. The initial feasibility of the guide was evaluated using an explanatory sequential mixed methods design, aligned with the early stages of Bowen's feasibility framework [45, 46], focusing on whether the guide ‘can work’ by gathering contextual insights on acceptability, demand and sensibility. Although the sample size was limited to five ‘Dance Teacher Reviewers’, purpose‐driven samples can provide valuable qualitative insights during early feasibility stages, particularly when iterative and participatory approaches are employed [46, 47].

Data were collected through a study specific online survey that captured both quantitative and qualitative data, followed by semi‐structured focus group [24, 46, 48]. The survey and focus group questions were designed by the research team and included feedback from the co‐design team, provided in Appendix S2. These questions were grounded in Bowen's feasibility framework, focusing on acceptability and demand [46]. Acceptability was measured by examining dance teachers' satisfaction with the guide, their intent to continue using it, and its perceived fit within their organisational culture. Demand was assessed through both the actual use of the guide, expressed interest in it, and the perceived need for the guide. Additionally, Feinstein's Evaluation of Sensibility model was used as a critical first step in evaluating the guide, covering multiple constructs such as the guide's format, face and content validity, and ease of use [48, 49]. Quantitative data were analysed using descriptive statistics to summarise participants' responses. Qualitative data were analysed using both inductive and deductive approaches. Deductive codes were generated from Bowen's framework to assess initial feasibility outcomes, whereas Feinstein's sensibility model further examined the guide's relevance, clarity and practical application for dance teachers [50].

## 3 Results

### 3.1 Phase 3

Six 90‐min online co‐design workshops were conducted between October 2023 and January 2024, including an internal review phase before sending the guide for external review (Phase 4). The co‐design team collectively endorsed the guide, confirming its readiness for external review. This collaborative effort resulted in the development of the co‐designed guide titled ‘Co‐Designed Inclusive Dance Approach Guide – Inclusion of Children with Cerebral Palsy’.

#### 3.1.1 Co‐Design Team Characteristics

Eleven people took part in the co‐design process, including parents supporting people with CP to engage in dance (*n* = 2), dance teachers (*n* = 2), health professionals (*n* = 2) and researchers (*n* = 5). While the guide is designed primarily for children with CP, perspectives from both children and parents were foundational to its development, particularly those gathered through interviews during Phase 2. The co‐design team included parents with diverse experiences, those who had advocated for their child's inclusion in community activities over many years and had finally found inclusion despite accessibility challenges and those currently supporting younger children navigating dance therapy while still seeking inclusion in community dance. These perspectives offered crucial insights into both long‐term advocacy and the immediate barriers to participation.

The dance teachers included a ballet dancer experienced in teaching dance to older individuals with disabilities and a studio dance teacher with experience in teaching dance to children 3–10 years old. The clinical team comprised a paediatric occupational therapist with experience in dance therapy and a dance movement therapist with a background in occupational therapy.

The academic team included five researchers with diverse backgrounds and expertise: J.N., a dance and exercise scientist with expertise in translating research findings to practitioners, industry and the community; E.D.M., the project lead and workshop facilitator, a physiotherapist with a background in child development and wheelchair dance programs; L.M., who provided facilitation support during the workshops, bringing 20 years of experience in paediatric rehabilitation, family engagement, CP and participation; M.C., an exercise scientist with expertise in biomechanics, neuroscience and high‐technology systems for assessing balance, mobility and neuromuscular function; and P.H., who contributed 30 years of experience leading motor and cognitive development projects. All researchers had prior experience with qualitative and mixed‐methods research, significantly enriching the co‐design process.

#### 3.1.2 Guide Format and Structure

**Figure 3:**
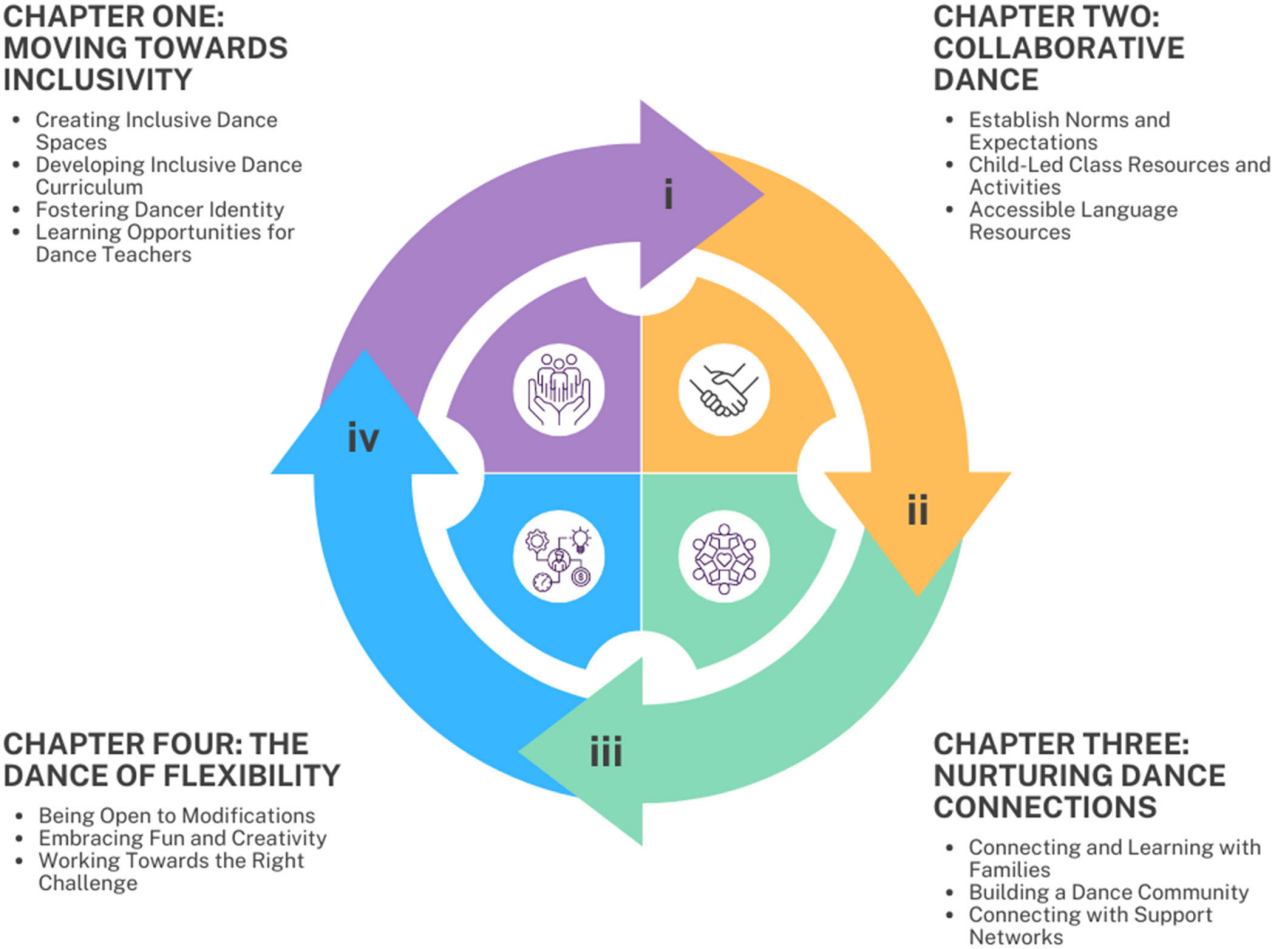
Diagram detailing the approach taken for each chapter of the guide and their interconnectedness.

The guide was designed with a user‐friendly PDF format, with written content, photos, and links to external resources, such as videos and websites. This approach enhances engagement, through easy navigation, and comprehension, accommodating diverse learning styles. It begins with an overview that explains the holistic and interconnected nature of each chapter. The guide is organised into four chapters that form a cohesive narrative toward a comprehensive understanding of inclusivity in dance practices, as illustrated in Figure 3.

Each chapter starts with a descriptive overview, providing background and context for the proposed strategies within an inclusive dance practice. The chapters end with a list of core and additional resources to support the implementation of these strategies. Table 1 provides a brief overview and examples of the guide. Importantly, the guide encourages users to adapt the strategies to their specific contexts, considering their experiences, practices and the needs of the children and families they work with.

**Table 1:** Overview of the ‘Co‐Designed Inclusive Dance Approach Guide – Inclusion of Children with Cerebral Palsy’ chapters and resources.

Chapter sections	Resources and strategies
**Chapter 1: Moving Toward Inclusivity**	
*Creating Inclusive Dance Spaces*	Environmental and structural changes to ensure inclusion, resources such as Accessible Arts Starter Kit are recommended.
*Developing Inclusive Dance Curriculum*	Methodologies aimed at fostering inclusion, such as Universal Design for Learning (UDL).
*Fostering Dancer Identity*	Tools and strategies to promote attitudinal changes and identity formation, such as videos of dancers with different abilities.
*Learning Opportunities for Dance Teachers*	Empowering dance teachers with knowledge and tools to promote inclusivity; podcasts, webinars and professional development are recommended.
**Chapter 2: Collaborative Dance**	
*Establish Norms and Expectations*	Establishing a culture of respect and acceptance, tools such as the F‐words framework and the Conversations for Collaboration framework are recommended.
*Child‐Led Class Resources and Activities*	Encouraging child‐led approaches and co‐creation. Conversations for Collaboration tools for student‐centred approach.
*Accessible Language Resources*	Improving communication to ensure inclusivity, videos with recommendations on communication and visual schedules
**Chapter 3: Nurturing Dance Connections**	
*Connecting and Learning with Families*	Focus is on fostering positive relationships between teachers and families: warm greetings, casual conversations, welcoming body language, regular email updates and relaxed interactions are effective strategies for relationship building.
*Building a Dance Community*	Early Years Learning Framework highlights the importance of fostering a sense of belonging and connection among children.
*Connecting with Support Networks*	The Conversations for Collaboration tools can be used to facilitate the communication between dance teachers and support staff.
**Theme 4: Flexibility Within Structure**	
*Being Open to Modifications*	Approaches such as CO‐OP, Layering, and the FAMME model will ensure dancers collaborate on a guided discovery of new movements and find their way to move.
*Embracing Fun and Creativity*	Integrating less structured exercises and embracing the play and joyful aspects of dance while exploring movement skills. Creativity is an important component in dance to foster one's sense of achievement and it is often associated with well‐being and success.
*Working Towards the Right Challenge*	Setting realistic goals and understanding talent in dance; considering physical, artistic and psychological factors. The Family of Participation‐Related Constructs (fPRC) can guide goal setting and challenge adjustment for individual dancers.

The content was co‐developed and refined by the co‐design team through an iterative process during the workshops. The lead author compiled and structured the guide based on the strategies identified, whereas co‐design team members offered ongoing feedback and endorsement, to ensure it accurately reflected their contributions and insights.

### 3.2 Phase 4

#### 3.2.1 Guide Review

Following the co‐design process, five ‘Dance Teacher Reviewers’, working in community‐based dance classes in Australia, provided survey data with feedback on the guide, whereas four (80%) of the reviewers also participated in a focus group. The fifth reviewer was unable to attend due to scheduling conflicts but had expressed their views comprehensively in the survey. Participant characteristics are presented in Table 2, with limited information provided to protect participants' identities. Reflective discussions during the focus groups, along with survey responses, highlighted the guide's acceptability, sensibility and demand. ‘Dance Teacher Reviewers’ found the guide to be both relevant and useful to their practices. In the survey, all participants indicated they would be likely to use the guide in their dance practice (Table 3). Additionally, four participants agreed, and one strongly agreed, that the guide's resources could be easily adapted and implemented within their organisations (Table 4), *‘I think overall, really good, and will be quite easy to implement’* (P2).

**Table 2:** Dance Teacher Reviewers' demographics.

Variables	Number
Professional experience	
School dance teacher	1
Ballet dance teacher	2
Tap dance teacher	1
Acrobat dance teacher	1
Australian Geographic Location	
Metropolitan	4
Regional	1
Years of experience	
< 10 years	2
> 10 years	3

**Table 3:** Dance Teacher Reviewers' use and adoption of the guide.

Yes/no questions	Yes (*N* = 5)	No (*N* = 5)
Would you need help in understanding how to use the resources?	0	5
Having been introduced to the guide, would you be likely to use the inclusive dance approach in your dance practice?	5	0
Do you already use some of the resources suggested in the guide in your dance practice to support inclusivity?	4	1

**Table 4:** Dance Teacher Reviewers' ratings on the guide's presentation, content and resources.

Statement	Strongly disagree *N* = 5	Disagree *N* = 5	No opinion *N* = 5	Agree *N* = 5	Strongly agree *N* = 5
**Presentation and content of the guide**					
The guide is relevant and useful for my dance practice.	0	0	0	4	1
The language used in the guide is easy to understand.	0	0	0	0	5
The format of the guide is comprehensive.	0	0	0	0	5
The format of the guide is easy to follow.	0	0	0	2	3
**Supporting resources and readings suggested in the guide**					
I could easily use the resources to improve inclusivity in my dance practice.	0	0	0	3	2
I could easily use the resources to plan and develop my activities.	0	0	1	3	1
I could easily use the tools to identify opportunities for dancers to engage in activities.	0	0	0	3	2
I could easily adapt and implement the resources.	0	0	0	4	1
I found the resources were helpful to assess the quality and impact of my initiatives.	0	0	1	2	2

#### 3.2.2 Demand

The need for the guide was evident both in the survey responses and themes that emerged from focus group discussions. All ‘Dance Teacher Reviewers’ expressed their intent to continue using the guide's resources, and four of them indicated in the survey (Table 4) they were already familiar with some of the suggested resources, such as the Universal Design for Learning (UDL) framework, layering strategies and elements of the Cognitive Orientation to daily Occupational Performance (CO‐OP) approach. One ‘Dance Teacher Reviewers’ was not familiar with any of the guides resources.

[…] for my kids at school next term, they do a whole unit on social dance […] and for some of my students who need additional needs that can create barriers for them to participate. So, I think those vision boards and brainstorming […] are definitely something I'll be, […], trying with some of those students to see how they work in application for them

(P3)

During the focus group, ‘Dance Teacher Reviewers’ also highlighted the importance of a training program to support the guide's implementation. They expressed that such a program would not only build their confidence in using the strategies and resources recommended by the guide but also foster a community of like‐minded professionals. The consensus was that such a training program would provide both the knowledge needed to effectively use the guide and a supportive network to sustain ongoing collaboration and development.

If you do a training course, you are usually with other people and then you get this network as well. […] any time you do a training, you should connect with somebody, because that's absolute gold to bounce ideas off. It's a like‐minded person that's doing the training as well for a reason, because it's obviously relevant to them

(P4)

#### 3.2.3 Acceptability of the Guide

All four of the ‘Dance Teacher Reviewers’ involved in the focus group expressed satisfaction with the guide, with one commenting. The perceived appropriateness of the guide's content was also reflected in the survey results, where two participants strongly agreed, and three agreed, that they could easily use the tools to identify opportunities for dancers to engage in activities (Table 4). During the focus group, participants highlighted that tools such as the F‐words framework would be easy to implement.

Coming from more of a studio context in a very, I would say, casual dance school, we're not very high performance that we don't see our students for more than maybe 40 min [to] an hour a week. I really liked the F‐words and the proformas and the goal setting

(P1)

Although all four dance teachers involved in the focus group agreed that the guide would align with their organisations' values, they identified a potential cultural barrier that could hinder its implementation. They expressed concern that parents of children without disabilities might pose the biggest challenge, as one participant mentioned.

I think that it [the barrier] wouldn't be the organisation, or the teachers, or even the other students in the class, or the parents who are bringing children with disabilities. It's going to be the parents whose child doesn't have a barrier and them feeling, whatever they're feeling, I think that would be the biggest concern.

(P3)

Although the guide was well‐received and deemed appropriate, ‘Dance Teacher Reviewers’ stated that addressing the ableist culture within the dance community and among families would be crucial for successful implementation.

#### 3.2.4 Guide Sensibility

All five ‘Dance Teacher Reviewers’ had a positive overall impression of the guide, they agreed the format of the guide was comprehensive and the language used in the guide was easy to understand (Table 4), *‘I thought it was really well set out, and I really enjoyed it.’* (P1). Despite this positive feedback, there were some recommendations for enhancing the guide's format, particularly in relation to navigation. ‘Dance Teacher Reviewers’ suggested incorporating solid‐coloured banners at the top of the pages to clearly delineate chapters, which would facilitate easier navigation.

The only thing in terms of coloured banners or the chapters […]. I found sometimes when I was scrolling between [chapters], […] I want to go back to Chapter 2, or I want to go down to Chapter 3, that's probably where I found sometimes getting a bit confused.

(P3)

Regarding the guide's content, reviewers found it highly relevant and comprehensive for supporting inclusive dance practices. During the focus group discussion, they suggested including examples or short vignettes to provide concrete ways to implement the guide's recommendations.

I teach with a lot of assistant teachers, who might not have as much experience or background with teaching kids, especially kids with additional needs. So, having really solid examples of like language use or questions to ask, I think would be really helpful for applying [it] with those assistant teachers as well as us teachers.

(P1)

Overall, the guide sensibility was praised for being easy to follow, with straightforward and easy to understand language and highly relevant and comprehensive content.

## 4 Discussion

The ‘Co‐Designed Inclusive Dance Approach Guide – Inclusion of Children with Cerebral Palsy’ was developed from a robust collaboration between knowledge users and researchers, employing a rigorous interactive methodology committed to co‐creation and PAR principles [19, 23, 31]. This approach is critical in developing tools that are both relevant and sustainable in real‐world contexts, ensuring that the outputs genuinely reflect the participants' needs and experiences [31, 51]. The guide addresses a significant gap in resources tailored to enhancing the participation of children with CP in community‐based dance classes, responding to a clear demand for inclusive practices [52, 53].

The guide seeks to meet the identified need for a practical, user‐friendly tool, aimed at enhancing the accessibility and fostering the participation of children with CP in community‐based dance classes. This aligns with the current shift toward inclusive practices and participation in community activities [10, 54, 55]. In Australia, the recent Disability Royal Commission (DRC) [11] proposed changes to laws, policies, structures and practices to foster a more inclusive and equitable society that supports the independence of people with disabilities. Among the 222 recommendations was the advocacy for transitioning toward inclusive education and implementing substantial reforms to the mainstream systems to eliminate barriers to access and promote meaningful inclusion [56].

In response to the disconnect between policy goals and real‐world outcomes, the guide contributes to workforce upskilling and the creation of inclusive environments in the dance sector, which has often been influenced by ableist culture [12], where societal norms and expectations prioritise able‐bodied individuals, emphasising conventional performance and traditional aesthetics over creativity, motivation and self‐confidence, limiting opportunities for children with CP to express themselves and develop alongside their peers [13, 57]. The guide advocates for inclusive spaces where children with disabilities can participate fully and shape the art form through their unique contributions, shifting the narrative from mere presence to meaningful engagement [58]. It also promotes the importance of placing dancers with disabilities, such as CP, in leadership roles to challenge societal attitudes and advance inclusion [29, 59]. By offering practical strategies and fostering cross‐sector partnerships, the guide addresses the need for structured training for dance teachers to adapt activities for children with CP [53, 60]. This aligns with broader calls for professional development to enhance inclusive practices and ensure children with CP can thrive in community activities [3, 61].

Furthermore, the guide was positively received by dance teachers, who found it highly relevant and useful for their practices. This positive reception is largely attributed to the guide's strong foundation in contemporary evidence from the paediatric healthcare and education sectors, drawing from well‐established theoretical frameworks such as the Self‐Determination Theory (SDT), the fPRC, the F‐words framework and the Early Years Learning Framework for Australia: Belonging, Being and Becoming [14, 62, 63, 64]. These frameworks are integral to the guide's design, ensuring that the tools provided are effective, evidence‐based and aligned with best practices in childhood development [53, 63, 65, 66].

The interconnectedness of the guide's chapters and tools facilitates ease of use and reproducibility across different contexts; thus, familiarity with one tool is easily translated to another. As highlighted by Morris et al. [61], factors such as enjoyment, relatedness, competence and autonomy are crucial in sustaining participation in physical activities among adolescents with CP. The guide's emphasis on these elements, particularly through the incorporation of the fPRC and the F‐words frameworks, promotes autonomy, individual strengths, social skills, creative ability and self‐confidence [65, 67]. This holistic approach, which also draws on contemporary concepts such as the expanded notion of health and functioning, aligns with the goals of inclusive education and community participation, highlighting the process of being, becoming and belonging [57, 67, 68].

### 4.1 Implications of Findings and Future Research

A significant strength of this study is the collaborative and participatory approach used to develop the guide, which ensured that it is both relevant and practical for knowledge users. The co‐design process, involving knowledge users such as children with CP, their families and dance teachers, ensured that the guide meets real‐world needs and addresses the practical challenges of fostering inclusive practices in community‐based dance programs.

The tools utilised in the guide were drawn from existing resources commonly found in the health and education sectors, despite the lack of specificity to dance and immediate familiarity to all dance teachers, the feedback from the ‘Dance Teacher Reviewers’ indicated that the guide was easy to understand and thought it could be implement across a variety of contexts. This cross‐sector collaboration ensures a more holistic approach, integrating the expertise of health professionals, educators and community leaders to create inclusive environments that are responsive to the unique needs of children with CP [65, 69]. This comprehensive approach to inclusion aligns with contemporary theories of disability, focusing on autonomy, social engagement and community participation.

Although review of the guide involved a relatively small sample of only five dance teachers, which may limit the generalisability of the findings, this initial phase provided valuable insights. The findings from this pilot review will inform further development of the guide in preparation for a more extensive evaluation of its effectiveness and impact. Future research should focus on a larger scale feasibility study involving a more diverse group of knowledge users, including families and children with CP, to evaluate the guide's acceptability, demand and sensibility [46]. This would enable a more comprehensive assessment of the guide and provide opportunities for meaningful refinements based on broader feedback.

## 5 Conclusions

The guide to supporting inclusive dance approaches described in this study is novel in several respects. First, it was developed in collaboration with knowledge users, including a range of dance teachers from different areas of dance education, families of children with CP, and children with lived experience. The multiphase nature of the study facilitated engagement with knowledge users from diverse backgrounds and levels of experience in dance classes, ensuring a richness in perspectives and enhancing the guide's relevance across diverse audiences. The workshops provided invaluable insights, and the research partners offered relevant experiences and skills to identify the practical tools embedded within the guide. Moving forward, the guide will be made freely available and shared widely with community‐based dance classes across Australia. We encourage those actively involved in promoting inclusive practice to pilot the guide and offer feedback, which will be instrumental in continually refining and enhancing its effectiveness. This ongoing feedback will also help identify gaps or resource needs, ensuring that the guide remains current through the regular revision of resources and incorporation of newly available research and tools.

## Author Contributions

**Eduardo Duarte Machado:** conceptualisation, investigation, methodology, validation, formal analysis, writing – original draft, writing – review and editing, data curation. **Laura Miller:** conceptualisation, supervision, data curation, writing – review and editing, methodology, validation, investigation, writing – original draft, formal analysis. **Joanna Nicholas:** conceptualisation, writing – review and editing, writing – original draft, formal analysis, validation. **Joanne Cross:** validation, writing – original draft, conceptualisation, formal analysis. **Rhyannon Orr:** validation, writing – original draft, conceptualisation, formal analysis, writing – review and editing. **Michael H. Cole:** conceptualisation, writing – original draft, formal analysis, validation, writing – review and editing, supervision, data curation, methodology, investigation.

## Ethics Statement

Ethical approval was obtained from the Australian Catholic University's Human Research Ethics Committee (HREC) (Application ID: 2021‐302H).

## Consent

Participants provided consent that data, including their words, may be quoted in publications, reports and other research outputs, but that their names would not be used, and their identity would always be kept anonymous. All participants provided written informed consent; children and youth who were younger than 18 years old provided informed assent.

## Conflicts of Interest

The authors declare no conflicts of interest.

## Supporting information

Appendix S1.

Appendix S2.

## Data Availability

The data that support the findings of this study are available from the corresponding author upon reasonable request and ethical approval.
