# Supplementary material for: Developing an Inclusive Dance Guide for Children With Cerebral Palsy: A Co‐Design Process and Initial Feasibility Study
Source: Health Expect. 2025 May 27;28(3):e70304. doi: 10.1111/hex.70304 (PMC12116933; doi:10.1111/hex.70304)
Supplement: Supplementary file 1 — Appendix S1. [file HEX-28-e70304-s002.docx]

**Appendix S1 – GRIPP2-SF**

| **Table 1. Description of knowledge users’ involvement using GRIPP2-Short Form (Staniszewska et al., 2017) reporting guidelines.** | |
| --- | --- |
| **Section and topic** | **Description** |
| 1. Aim: Report the aim of PPI in the study | To ensure the research is meaningful, useful, appropriate to young people with cerebral palsy (CP).  To increase the quality and credibility of the study by integrating comprehensive knowledge of the lived experience of those with CP and dance teachers into the research processes.  To promote uptake of the research and translation of the work into practice through involvement of end users |
| 2. Methods: Provide a clear description of the methods used for PPI in the study | A Consumer Advisory Panel (CAP) was created to formally involve knowledge users throughout all phases of the research process. Recruitment was facilitated through existing networks, such as dance and research groups, as well as community organisations, which extended the invitation to their broader communities. The voices of the CAP were prioritised throughout and integrated into decision making to increase the authenticity of knowledge user involvement in the research. The CAP included: (i) two parents supporting people with CP to engage in dance; (ii) a ballet dancer with experience in teaching dance to people with disabilities; (iii) two allied health therapists with experience in dance interventions; and (iv) a dance researcher. |
| 3. Study results: Outcomes―Report the results of PPI in the study, including both positive and negative outcomes | The CAP was involved in the development and implementation of the research. The CAP played an important role in the focus group phase and their involvement shaped methodological decisions and data interpretation. In the co-design phase, knowledge users actively contributed to the development of the guide by finding resources, reviewing the guide, and providing input into the guide content, including attending to implicit messages in images and text. Each process and step can be time consuming and not all members of the CAP were involved as fully as they desired due time and other personal commitments. Flexibility with involvement was provided and knowledge users’ preferences, access needs and availability were determined using tools such as the Family Engagement in Research (FER) Goal Setting tool, the Involvement Matrix, and a team charter, to overcome challenges with time management. |
| 4. Discussion and conclusions: Outcomes―Comment on the extent to which PPI influenced the study overall. Describe positive and negative effects | The co-design process resulted in a guide that was more strength-based, the knowledge user research partners influenced the content of the guide, the delivery and usability processes, and the decisions about what to research next. The CAP involvement throughout the research lifecycle enhanced study quality, relevance, involvement of participants in interviews and focus groups, and data interpretation. |
| 5. Reflections/critical perspective: Comment critically on the study, reflecting on the things that went well and those that did not, so others can learn from this experience. | Involving a Consumer Advisory Panel optimised knowledge users’ engagement in each phase of the research. A good working relationship was established between the members of the CAP and the research team that enabled sustained involvement. The research would have benefited by earlier involvement of knowledge users to direct the course of the research from the perspectives of people with lived experience from the outset. For example, the partnership with knowledge users resulted in a shift from research questions focusing on participation in dance interventions to a broader approach by co-creating a practical guide for inclusive dance in community practices. Overall, the changes throughout the study reflect the successful partnership with knowledge users. Future studies must allocate appropriate time to remunerate knowledge users accurately to reflect their time and dedication to the project. |

PPI, patient and public involvement.

**References**

Staniszewska, S., Brett, J., Simera, I., Seers, K., Mockford, C., Goodlad, S., Altman, D. G., Moher, D., Barber, R., Denegri, S., Entwistle, A., Littlejohns, P., Morris, C., Suleman, R., Thomas, V., & Tysall, C. (2017). GRIPP2 reporting checklists: tools to improve reporting of patient and public involvement in research. *BMJ (Online)*, *358*, j3453-j3453. <https://doi.org/10.1136/bmj.j3453>
