# Supplementary material for: Developing an Inclusive Dance Guide for Children With Cerebral Palsy: A Co‐Design Process and Initial Feasibility Study
Source: Health Expect. 2025 May 27;28(3):e70304. doi: 10.1111/hex.70304 (PMC12116933; doi:10.1111/hex.70304)
Supplement: Supplementary file 2 — Appendix S2. [file HEX-28-e70304-s001.docx]

**Appendix S2a – Survey questions**

User consultation on the “Co-Designed Inclusive Dance Approach Guide”

Demographic questions

1. What genre of dance do you work with?
2. What age group do you work with?
3. What is your geographic location: Remote; Regional or Urban?
4. How many years of experience do you have teaching dance?

5-point Likert-scale questions

1. The following statements are about the presentation and content of the Inclusive Dance Approach Guide. Please read each statement and choose the option that aligns best with your opinion.

[Strongly Disagree/ Disagree/ No opinion/ Agree/ Strongly Agree]

- 1. The guide is relevant and useful for my dance practice.
  2. The language used in the guide is easy to understand.
  3. The format of the guide is comprehensive.
  4. The format of the guide is easy to follow.

1. If you have specific comments regarding the presentation and content of the Inclusive Dance Approach Guide, please share them here (optional):
2. The following statements are about the supporting resources and readings suggested in the guide. Please read each statement and choose the option that aligns best with your opinion.

[Strongly Disagree/ Disagree/ No opinion/ Agree/ Strongly Agree]

- 1. I could easily use the resources to improve inclusivity in my dance practice.
  2. I could easily use the resources to plan and develop my activities.
  3. I could easily use the tools to identify opportunities for dancers to engage in activities.
  4. I could easily adapt and implement the resources in my dance practice.
  5. I found the resources were helpful to assess the quality and impact of my initiatives.

1. If you have specific comments regarding the supporting resources and readings suggested in the guide, please share them here (optional):
2. Would you need help in understanding how to use the resources?

No or Yes → If yes, please specify what kind of help you would need. _____________

1. Having been introduced to the Guide, would you be likely to use the inclusive dance approach in your dance practice?

Yes or No → If not, why not? _________________

1. Do you already use some of the resources suggested in the Guide in your dance practice to support inclusivity?

No or Yes → If yes, please specify which tool(s) you use ____________

Detailed feedback (optional)

If you have specific comments for the “Co-Designed Inclusive Dance Approach Guide”, please share them here: (suggestions / amendments to the current guide, structure, language, examples...). This question is optional.

**Appendix S2b – Focus Group questions**
Our focus group aims to see how well the Inclusive Dance Approach Guide works for dance teachers. We want to understand if it fits well with their needs, if it’s relevant, useful, easy to use, and practical. We also want to see how acceptable, needed, easy to implement, practical, and adaptable the guide is. This will help us get detailed feedback on how effective and user-friendly the guide is in real dance settings.

1. In the survey it was suggested to have solid-coloured banners at the top of each chapter. How do you feel about this suggestion? Do you think it would help in navigating the guide more easily?
   1. Regarding the guide's visual aids (images, tables, infographics), how effective were they in enhancing your understanding?
   2. Would it be helpful to see resources used in context, such as in lesson plans or through case studies?
2. How clear and appropriate did you find the language used in the Guide?
   1. Were there specific terms or sections that were difficult to understand?
   2. Are there other ways you believe the guide's structure could be improved to enhance comprehension?
3. What aspects of the guide do you find relevant to your current dance practice?
   1. What additional content or examples would make the guide more relevant to your practice?
4. In what ways do you find the guide useful for your dance practice?
   1. Can you share specific examples of where the guide would be particularly helpful or less applicable?
5. What resources (time, materials, training) do you feel are necessary to effectively implement the Inclusive Dance Approach?
   1. Can you describe how you would adapt your dance classes using the recommendations from the guide?
   2. What additional support or guidance would you need to fully implement the inclusive dance strategies in your practice?
   3. Managing fatigue in children with CP has been identified as a significant concern. Based on the recommendations in the guide, what specific strategies would you suggest for effectively managing this challenge in a dance class setting?
6. From your experience, how receptive do you think parents and children will be to the Inclusive Dance Approach? What concerns or barriers do you think they might have?
   1. Are there any cultural attitudes towards dance and disability or organisational barriers within your dance school or community that might impact the acceptance and implementation of the Inclusive Dance Approach Guide?
   2. What strategies could be employed to overcome these barriers and improve acceptance?

Based on your experience, what further improvements or changes would you suggest for the Inclusive Dance Approach Guide?
